# Identifying preclinical vascular dementia in symptomatic small vessel disease using MRI

**DOI:** 10.1016/j.nicl.2018.06.023

**Published:** 2018-06-20

**Authors:** Christian Lambert, Eva Zeestraten, Owen Williams, Philip Benjamin, Andrew J. Lawrence, Robin G. Morris, Andrew D. Mackinnon, Thomas R. Barrick, Hugh S. Markus

**Affiliations:** a Wellcome Centre for Human Neuroimaging, 12 Queen Square, WC1N 3BG London, UK; b Neurosciences Research Centre, Molecular and Clinical Sciences Research Institute, St George's University of London, SW17 0RE, UK; c Stroke Research Group, Clinical Neurosciences, University of Cambridge, CB2 0QQ, UK; d Department of Psychology, King's College Institute of Psychiatry, Psychology, and Neuroscience, London, UK; e St George's NHS Healthcare Trust, Atkinson Morley Regional Neuroscience Centre, London, UK

**Keywords:** Structural MRI, Voxel-based morphometry, Vascular dementia, Preclinical dementia, Cerebral small vessel disease

## Abstract

Sporadic cerebral small vessel disease is an important cause of vascular dementia, a syndrome of cognitive impairment together with vascular brain damage. At post-mortem pure vascular dementia is rare, with evidence of co-existing Alzheimer's disease pathology in 95% of cases. This work used MRI to characterize structural abnormalities during the preclinical phase of vascular dementia in symptomatic small vessel disease.

121 subjects were recruited into the St George's Cognition and Neuroimaging in Stroke study and followed up longitudinally for five years. Over this period 22 individuals converted to dementia. Using voxel-based morphometry, we found structural abnormalities present at baseline in those with preclinical dementia, with reduced grey matter density in the left striatum and hippocampus, and more white matter hyperintensities in the frontal white-matter. The lacunar data revealed that some of these abnormalities may be due to lesions within the striatum and centrum semiovale.

Using support vector machines, future dementia could be best predicted using hippocampal and striatal Jacobian determinant data, achieving a balanced classification accuracy of 73%. Using cluster ward linkage we identified four anatomical subtypes. Successful predictions were restricted to groups with lower levels of vascular damage. The subgroup that could not be predicted were younger, further from conversion, had the highest levels of vascular damage, with milder cognitive impairment at baseline but more rapid deterioration in processing speed and executive function, consistent with a primary vascular dementia. In contrast, the remaining groups had decreasing levels of vascular damage and increasing memory impairment consistent with progressively more Alzheimer's-like pathology. Voxel-wise rates of hippocampal atrophy supported these distinctions, with the vascular group closely resembling the non-dementing cohort, whereas the Alzheimer's like group demonstrated global hippocampal atrophy.

This work reveals distinct anatomical endophenotypes in preclinical vascular dementia, forming a spectrum between vascular and Alzheimer's like pathology. The latter group can be identified using baseline MRI, with 73% converting within 5 years. It was not possible to predict the vascular dominant dementia subgroup, however 19% of negative predictions with high levels of vascular disease would ultimately develop dementia. It may be that techniques more sensitive to white matter damage, such as diffusion weighted imaging, may prove more useful for this vascular dominant subgroup in the future.

This work provides a way to accurately stratify patients using a baseline MRI scan, and has utility in future clinical trials designed to slow or prevent the onset of dementia in these high-risk cohorts.

## Highlights

• Preclinical vascular dementia is associated with structural changes.
• Using MRI, 73% who later develop vascular dementia are identified.
• Dementia subgroups are identified using anatomical patterns of damage.
• A spectrum between pure vascular and Alzheimer's-like patterns is found.
• Successful predictions of future dementia map to the Alzheimer's groups.

## 1 Introduction

It is generally accepted that future treatments for dementia should aim to start prior to symptom onset, when extensive pathology will already be present and difficult to reverse (Langbaum et al., 2013). Therefore accurately identifying individuals during the preclinical phase of their illness is paramount to developing effective therapies. Vascular cognitive impairment is defined as a syndrome in which at least one cognitive domain is impaired, together with evidence of vascular damage (Thal et al., 2012), and becomes vascular dementia once a second cognitive domain becomes affected (Gold et al., 1997). This clinically heterogeneous entity is the second most common cause of dementia after Alzheimer's disease (Jellinger and Attems, 2010), and can manifest due to a broad range of sporadic and familial conditions where the net result is vascular damage to the brain. The most common cause is cerebral small vessel disease (SVD), a highly prevalent, age-related condition affecting the small vessels of the brain (De Leeuw et al., 2001; Pantoni, 2010) that is associated with characteristic changes on magnetic resonance imaging (MRI) including white matter hyperintensities (WMH), lacunar infarcts, cerebral microbleeds, and brain atrophy (Gouw et al., 2011).

This work examines, in a cohort followed for 5 years, the structural correlates of preclinical vascular dementia using MRI to predict the development of future dementia in a cohort of individuals with sporadic “*symptomatic small vessel disease*”, defined as both clinical and radiological evidence of a lacunar syndrome together with confluent WMH (modified Fazekas grade ≥ 2), but without evidence of large cortical infarcts, cerebral amyloid angiopathy or other cause of white matter disease.

### 1.1 Dementia in cerebral small vessel disease – pathology

The most common pattern of pathology observed in vascular dementia is a subcortical vascular encephalopathy. This is used to describe a severe form of SVD due to arteriolosclerosis and lipohyalinosis (Ferrer, 2010; Thal et al., 2012), appearing as confluent leukoaraiosis on brain imaging with sparing of the cortico-cortical u-fibers (Jellinger and Attems, 2010). It is thought that the white matter damage in leukoaraiosis leads to dementia by way of a progressive disconnection syndrome due to damage of the cortical-subcortical and cortical-cortical connections (Lawrence et al., 2013). In addition single strategic infarcts may also cause or contribute to dementia when structures normally involved in mediating cognitive processes are damaged, for example the thalamus (particularly the paramedian or medio-dorsal thalamic nuclei) and hippocampi (Ferrer, 2010; Benjamin et al., 2014). However it should be noted that pure vascular dementia is rare at post-mortem, performed predominantly in elderly individuals, with histological evidence of co-existing Alzheimer's disease pathology present in 90–95% of cases (Jellinger and Attems, 2010; Thal et al., 2012).

### 1.2 Dementia in cerebral small vessel disease – clinical

The characteristic cognitive pattern associated the subcortical white-matter disease caused by SVD is that of prominent executive function and processing speed dysfunction, with relative preservation of episodic memory (Lawrence et al., 2013). In contrast, Alzheimer's disease and the mixed dementia subtypes are typified by more marked memory involvement (Reed et al., 2007).

### 1.3 Dementia in cerebral small vessel disease – MRI correlates

Whilst cognitive impairment in SVD has been shown to associate with a number of different MRI features including lacunar infarcts, WMH, and less consistently microbleeds (Patel and Markus, 2011; Benjamin et al., 2014), the imaging correlates of vascular dementia are less well defined. The most consistent finding is increased deep white matter hyperintensities (Smith et al., 2016; Altamura et al., 2016), associated with measures of reduced integrity of the white matter structural network (Tuladhar et al., 2016), which in combination have been found to be predictive of progression to future dementia. Whilst these previous MRI studies have not distinguished between vascular dementia subtypes, PET imaging using Pittsburgh compound B (PiB) to detect the β-amyloid protein has allowed the spectrum between the pure vascular, mixed and pure Alzheimer's dementias to be better characterized (Lee et al., 2011). These studies have found that individuals with pure vascular dementia are younger with substantially more lacunar infarcts (Lee et al., 2011; Kim et al., 2014) compared to those with evidence of co-existing β-amyloid pathology.

### 1.4 Hypotheses

Here we test the hypothesis that, within a cohort of individuals with symptomatic small vessel disease, there are particular localized structural abnormalities in those destined to convert to dementia within five years (“*preclinical vascular dementia*”) that can be identified from the baseline structural imaging (T1-weighted and FLAIR MRI). Furthermore, simple machine learning techniques (i.e. support vector machines) can be used to predict future dementia from the baseline MRI imaging. We also test the hypothesis that vascular dementia is associated with differences in the voxel-wise rate of cortical atrophy or WMH expansion. Finally, due to the known heterogeneous nature of vascular dementia, we aimed to use the structural imaging to define whether distinct anatomical endophenotypes exist within the preclinical cohort, and if so, were there any differences in the clinical manifestation or disease progression between these groups.

## 2 Methods

### 2.1 Subjects

Supplementary Fig. 1 summarizes the baseline and longitudinal data used, conversion to dementia and reasons for dropout. This work primarily focused on stratifying preclinical dementia based on the initial baseline assessment, and therefore included all individuals irrespective of their long-term outcome.

### 2.2 Subjects - baseline

121 subjects (78 male, mean age male = 67.96 ± 10 years, female = 73.74 ± 8.12 years) with symptomatic SVD were recruited as part of the prospective St George's Cognition and Neuroimaging in Stroke (SCANS) study (Lawrence et al., 2013). Recruitment was from acute stroke units or outpatient stroke clinics in three hospitals covering a contiguous catchment area in South London (St George's, King's College and St Thomas' Hospitals). Inclusion criteria comprised a clinical lacunar syndrome (Bamford et al., 1987) with an anatomically corresponding lacunar infarct in addition confluent WMH on MRI (modified Fazekas grade ≥2) (Fazekas et al., 1987; Hassan et al., 2003) on MRI. Exclusion criteria were: any cause of stroke mechanism other than SVD, other major central nervous system disorders, major psychiatric disorders, any other cause of white matter disease, contraindications to MRI, or non-fluent in English. All subjects provided written consent, and the study was approved by the local ethics committee. The study is registered with UK Clinical Research Network (http://public.ukcrn.org.uk/, study ID:4577). The T1-weigthed MRI for two of these individuals was corrupted by artifact (see Lambert et al., 2015) that resulted in very inaccurate tissue segmentations and therefore were excluded from this work. The remaining 119 individuals were used for all baseline analysis.

### 2.3 Subjects – longitudinal

Subjects were invited for cognitive testing and clinical assessment annually for five years. During the first three years they also underwent annual MRI scanning. Recruitment began in December 2007 and ended in August 2010. MRI scanning began in January 2008 and was completed in October 2013. If a participant was diagnosed with dementia at any point over the five years, they were allocated to the preclinical vascular dementia (PreVaD) baseline cohort.

In the MRI longitudinal rate analysis, follow-up data up to year three was used (i.e. the complete longitudinal MRI dataset), providing a maximum of four datasets per individual. Of these, 99 subjects returned at one or more time-points: 98 at year one, 77 at year two and 71 at year three. One subject attended the baseline and missed the year one follow up, but attended all subsequent sessions. Four subjects missed the year two follow up, but subsequently attended at year three. Additionally, over the period reported there were three new clinical strokes, two lacunar and one cortical haemorrhage. Intracerebral haemorrhage was a pre-defined end-point and the patient was withdrawn from the study. The two lacunar stroke cases were allowed to remain in the study, as stated in the protocol, although one subsequently withdrew due to disability. This is summarized in Supplementary Fig. 1. All available longitudinal data (n = 99) was used in the rate analysis, of whom 17 ultimately developed dementia.

### 2.4 Conversion to dementia - definition

Information on conversion to dementia was available for all patients. Dementia was diagnosed using the “Diagnostic and Statistical Manual of Mental Disorders V" (DSM–V) (American Psychiatric Association, 2013) definition of “*major neurocognitive disorder*”, and was present if individuals met one of the following criteria:

1. A diagnosis of dementia made in a memory clinic or equivalent clinical service.
2. After review of medical records and cognitive assessments by a neurologist and clinical neuropsychologist who were both blind to all MRI and risk factor information and who both agreed that the clinical picture met DSM-V criteria for dementia.
3. An MMSE score consistently <24, indicative of cognitive impairment (Tombaugh and McIntyre, 1992) and reduced capabilities in daily living as measured by a score ≤ 7 on the iADL (Barberger-Gateau et al., 1992).

In all cases the presence or absence of dementia was determined before comparison of the cognitive and MRI data. Date of dementia onset was defined as the date of diagnoses. If no exact date was known and dementia conversion was based on review of patient data or cognitive performance, the mid-point date between the patient's visit at which the diagnosis was established and the previous visit was used. If a participant was diagnosed with dementia at any point over the five years following recruitment, they were allocated to the preclinical vascular dementia (PreVaD) baseline cohort. No participant met the clinical DSM V criteria for vascular dementia at the time of recruitment, though one individual met research criteria at the baseline assessment. We elected to include this individual; on the basis we were testing what could be inferred from baseline imaging data alone.

### 2.5 Image acquisition

All subject MR images were acquired using the Signa HDxt 1.5 T Magnetic Resonance Scanner (General Electric, Milwaukee, WI, USA) at St George's, University of London. The maximum gradient amplitude was 33mTm^−1^ and a proprietary 8-channel head coil was used. All subjects were placed in the head coil and an alignment marker was used at the nasal bridge. Velcro straps and foam pads were used where possible to minimize head movement. Whole brain T1-weighted and FLAIR images were acquired for each subject using the following protocol: (1) Fluid Attenuated Inversion Recovery (FLAIR) sequence - TR/TE/TI = 9000/130/2200 ms, field-of-view (FOV) = 240 × 240 mm^2^, matrix = 256 × 192, 28 axial slices of 5 mm thickness reconstructing to a final image resolution of 0.47 × 0.47 × 5 mm^3^. (2) Spoiled gradient echo recalled T1-weighted (SPGR) 3D coronal sequence - TR/TE = 11.5/5 ms, FOV = 240 × 240 mm^2^, matrix = 256 × 192, flip angle = 18^o^, 176 coronal slices of 1.1 mm thickness reconstructing to a final image isotropic resolution of 1.1 mm.

### 2.6 Pre-processing

The raw DICOMS were imported using the SPM8 software package (http://www.fil.ion.ucl.ac.uk/spm/software/spm8/), and each image checked to ensure a common orientation. Initially the T1-weighted and FLAIR images were co-registered together using an affine transformation in SPM for each individual, before rigid transformation to the same orientation as the MNI template and resliced to 1 mm isotropic resolution using 4th degree b-spline interpolation.

### 2.7 Segmentation

The segmentation steps adapted and optimized to our study population have been described in detail in Lambert et al., 2015 and Lambert et al., 2016a, but are summarized below and a full description provided in the supplementary material (Supplementary Material). The adapted segmentation pipeline consisted of four steps. First, a group average template was created and non-linear warps used to transform the T1-weighted and FLAIR images to this space. Second, the warped T1-weighted and FLAIR images were used to create population specific tissue probability maps (TPMs). Third, the newly created TPMs were used to re-segment the native images creating grey matter (GM), white matter (WM), cerebrospinal fluid (CSF) and WMH tissue classes. These were then combined with the manually defined lacune ROIs (detailed below), resulting in five tissue classes per individual. Finally, a tissue repair step was performed to generate repaired GM, WM and CSF maps for each individual dataset. The VBCT toolbox (Hutton et al., 2008) in SPM8 was used to calculate cortical thickness (CT) using the final repaired segmentations with a sampling resolution of 0.5 mm, CSF smoothness 3 mm, CSF thinness 0.65 and number of dilations was set to one.

### 2.8 Lacune delineation

Areas of lacunar infarction were manually identified on native space T1-weighted images using published criteria (Benjamin et al., 2014; Lambert et al., 2015). This was aided by overlaying CSF segmentations on corresponding FLAIR images to identify regions of misclassification. Regions were segmented using a space-filling algorithm in ITK-SNAP (Yushkevich et al., 2006), using voxel intensity thresholds (set to between 350 and 500) with 500 iterations. These results were visually checked and manually refined where necessary. This step was performed independently by two raters (CL and PB), and inter- and intra-rater reliability checked using 10 randomly selected scans across all time points, duplicated to provide a total set of 20 scans per rater, each set randomly re-ordered for each rater to avoid any sequence bias. Both raters were blinded with respect to subject and time-point of each scan. The inter-rater reliability metrics were: Standard error of mean = 3 mm^3^, mean variability = 7.93% (standard deviation = 4.89%), Pearson's intra-class correlation coefficient = 0.99. The corresponding intra-rater reliability metrics were: Standard error of mean = 2 mm^3^, mean variability = 4.32% (standard deviation = 4.19%), Pearson's intraclass correlation coefficient = 0.99. There were no significant differences between the volumes measured by the two raters.

### 2.9 Cerebral microbleeds

CMB were defined as homogeneous round focal areas <10 mm in diameter of low signal intensity on T2*-weighted GRE images. Only CMB meeting the Brain Observer Microbleed Rating Scale (BOMBS) (Cordonnier et al., 2009) criteria for “certain” CMB were analyzed. Presence and number of CMB at baseline were identified by a single consultant neuroradiologist. CMB number reliability metrics were checked using a subset of 10 randomly selected subjects. The intra-class correlation coefficient was 0.99.

### 2.10 Baseline warping pipeline

All baseline repaired segmentation maps were visually checked for quality in native space and then warped to a final 1 mm isotropic group average template using a diffeomorphic-warping algorithm (the *Shoot* toolbox in SPM12 (Ashburner and Friston, 2011)) to generate an optimized group average image for further analysis. Using the same approach, the WMH maps were also warped to their own group average space.

### 2.11 Longitudinal warping pipeline

To calculate longitudinal volumetric changes in small vessel disease and to enable statistical analysis regardless of the number of time-points, we developed a two stage-warping pipeline by adapting the available SPM framework (described in detail in Lambert et al., 2016a, Lambert et al., 2016b), also provided in the Supplementary material) that first warped each subject to an individual average template that represents the average mid-point brain. This step provides a voxel wise trajectory, based upon the non-linear deformations to the average individual brain, for each time-point. These are expressed as divergence maps (for each time-point) in SPM as detailed below. To create a “*rate map*” we performed a voxel-wise linear fit between the divergence maps, using the time between scans as the basis (as per Ashburner and Ridgway, 2012). These individual rate maps show the relative speed of tissue expansion or contraction for every voxel in the brain per year and were used for analysis. For each subject, the following images from individual average space were warped to the group average template: Grey matter template, white matter template, WMH template, lesion rate maps and repaired rate maps. Each of the warped tissue classes was multiplied by the warped rate maps.

### 2.12 Whole brain MRI parameters

For each scan, the unrepaired segmentations were used to calculate tissue volumes. The lacunar infarcts, total cerebral volume (TCV, defined as the sum of grey matter, white matter and WMH at a tissue probability threshold ≥0.2) and total intracranial volume (TIV, defined as TCV plus the CSF volume at a set tissue probability threshold ≥0.2, constrained by the final brain inclusion mask) were calculated in mm^3^. The WMH volumes were calculated by binarising the segmentations at a manually determined threshold for each individual as detailed in the segmentation methodology (Supplementary material). We also calculated the ratio of WMH to the TCV (SVDp). This measure combines atrophy and WMH as a measure of severity, and prevents erroneous regression of WMH that may occur with zero WMH growth occurring against a background of continuing brain atrophy. For each longitudinal dataset, the annualized rate of change was calculated from all available time points (e.g. time in years from baseline) using a least squares linear fit in MATLAB 2013a. All MRI parameters were summarized according dementia convertors and non-convertors.

### 2.13 Cognitive assessment

A battery of well-established, standardised tasks sensitive to the cognitive impairments seen in SVD was carried out annually. Full details have been published previously (Lawrence et al., 2013), and are summarized in Supplementary Table 1. In brief, premorbid intelligence was assessed using the National Adult Reading Test-restandardised (NART) (Nelson and Willison, 1991) and the MMSE was used as a dementia screening tool. All other tasks were age-scaled using published normative data, converted to z-scores and grouped into broad cognitive domains. Averaging across component scores within each cognitive domain created four cognitive index scores (Supplementary Table 1): Executive function (EF), processing speed (PS), working memory (WM) and long-term (episodic) memory (LTM). An overall global functioning score based on all administered tests was also produced. The cognitive index scores were based on the following tasks: Executive function – Trail making test part B (Reitan, 1958), phonemic fluency (Delis et al., 2001), and the modified Wisconsin card-sorting test (Nagahama et al., 2003). Processing speed – Digit symbol substitution (Wechsler, 1997), the grooved pegboard task (Klove, 1963; Mitrushina et al., 2005), and the BMIPB speed of information processing speed test (Coughlan et al., 2007). Working Memory – Digit span (Wechsler, 1997). Long Term Memory – Logical memory and visual reproduction (Wechsler, 1997).

### 2.14 Statistical data analysis

All analysis was performed in MATLAB 2013a unless specified, and the data was grouped either according to time-point or outcome as specified. The normally distributed data was explored initially using an ANOVA, and if significant a two-sample *t*-test was used to examine differences between groups. Non-parametric data was tested using a Kruskal-Wallis test. Results significant at P < 0.05 were reported.

### 2.15 Lacunar infarct analysis

All lacunar infarct maps were warped to the group average space and used to create overlap maps for the two groups. To assess the differences between the two groups, a third map was created that identified regions where there were more infarcts present in the dementia group compared to the normal group. To assess the distribution of the lacunar infarcts, the following atlases were warped to the population average space using FNIRT in FSL: The Harvard-Oxford Subcortical Atlas (21 subcortical labels, available in FSL), the ICBM-DTI-81 white-matter labels atlas (48 white matter labels, available in FSL), tractography segmented thalamic nuclei (9 nuclei, taken from Lambert et al., 2016b). For each region of interest, the percentage of individuals where a lacunar infarct was present (and more than 10mm^3^ (Benjamin et al., 2014)) were calculated. These results are summarized in Supplementary Table 1.

### 2.16 Baseline statistical image analysis

All statistical image analysis was performed in SPM12. The baseline segmentations for GM, WM and WMH were warped to their group average space, modulated by multiplying by the Jacobian determinant, and smoothed using a 6 mm FWHM Gaussian kernel. The warped CT maps were used to generate FWHM 6 mm smoothed warped weighted average (WWA) images produced as described by Draganski et al., 2011 (Hutton et al., 2009; Draganski et al., 2011). These were all analyzed using a two-sample *t*-test in SPM to test for structural differences between those destined to convert to dementia versus those not. Age, gender, lacune infarct volume, TIV and NART were included as covariates. Regions that survived FWE multiple comparison correction at P < 0.05 were deemed significant. To better explore the FWE significant regions, they are displayed at both P < 0.001 uncorrected and P < 0.05 FWE corrected for multiple comparisons in the figures and appropriate legends provided.

### 2.17 Longitudinal analysis

#### 2.17.1 Longitudinal MRI and cognitive parameters

Linear mixed effect (LME) models to estimate annualized change rates in cognitive indices based on all available time-points using MLwiN 2.1 (Centre for Multilevel Modelling, University of Bristol (Rasbash et al., 2009). Random effect slopes for each patient as estimated by the LME models were extracted and used for further analyses. This data was used to assess the baseline anatomical clustering described below.

#### 2.17.2 Longitudinal statistical image analysis

The objective was to test for voxel wise differences in the rates of GM atrophy or WMH growth between the convertors and non-convertors. WWA rate maps were generated for the GM and WMH as previously described, and analyzed using a two-sample t-test in SPM12 to test for structural differences between those destined to convert to dementia versus those not as described above.

### 2.18 Prediction analysis

To further investigate the relationship between the MRI changes and dementia, we attempted to predict conversion from the baseline structural imaging data using a kernel based support vector machine (SVM) from the PRoNTo v2.0 toolbox (http://www.mlnl.cs.ucl.ac.uk/pronto/) (Schrouff et al., 2013). The same settings were used for all analysis: Binary SVM, age, sex, TIV and NART included as potential confounders, default hyperparameters, leave-one-out cross validation, mean centre features using training data, permutation test using 10,000 permutations. The following structural maps were separately tested: Jacobian determinant image, warped lacunar map, warped modulated WMH, smoothed warped weighted-average CT maps and the smoothed warped modulated GM and WM. To clarify the subcortical contribution, SVM predictions were also tested using masked Jacobian determinant images. The bilateral striatum (putamen and caudate), hippocampi and thalamic masks from the AAL atlas (provided in the PRoNTo toolbox) were used. If significant at *P* < .05, prediction quality was quantitatively assessed across a range of parameters including total classification accuracy, balanced classification accuracy, sensitivity, specificity, positive predictive value, negative predictive value and area under the receiver operator curve.

### 2.19 Cluster analysis

The Jacobian determinant images that had been generated from warps using the GM, WM, WMH, LI and CSF were used to characterize the structural similarities between the dementia cohort using cluster ward linkage. Initially, these were masked using a skull-striped brain mask and all voxels within this were extracted and used to generate a 22 × N matrix of Jacobian determinant values (where N = total brain volume). From this, a 22 × 22 correlation matrix was created and used to calculate the Euclidean distance between each individual. Ward-linkage was then used to generate a dendrogram, to visualise these similarities, and the prediction accuracies were superimposed. Four groups were selected based on visual inspection of the dendrogram and explored further. Due to the small numbers within each group formal between-group statistics were not possible, however to better understand nature of the sub-groups the corresponding demographic, MRI and cognitive parameters were extracted and summarized. To better understand the anatomical basis of the group differences, the average rate of hippocampal atrophy was calculated from the rate maps.

### 2.20 Survival analysis

Kaplan-Meier survival curves between the positive and negative predictions and time to dementia onset were generated using MATLAB. These were also further stratified by examining the impact of vascular burden (defined as a SVDp of more or <4%) on the SVM prediction survival curves. Differences between the survival curves were tested using the log-rank test (using “logrank” from the MATLAB file exchange: http://www.mathworks.com/matlabcentral/fileexchange/20388-logrank/content/logrank.m).

## 3 Results

### 3.1 Baseline demographics

Out of the baseline cohort (n = 119), 22 individuals (18.5%) developed dementia during follow-up with a mean time to conversion of 3.02 ± 1.50 years; we now refer to these as the preclinical vascular dementia cohort (preVaD). The demographics, cognitive and MRI parameters for these groups are summarized in Table 1. The only significant difference at baseline between those who did and did not convert to dementia at baseline after Bonferroni correction was the MMSE (Median (range): Normal = 29 (22−30), PreVaD = 26 (16–30)).

**Table 1:** Baseline demographics and parameters for cohort. All values are mean (standard deviation) unless labelled otherwise.

		Cohort	
	BASELINE PARAMETERS	Future non-dementia	Future dementia
Drmographics	N	97	22
	Average age (years)	69.49 (9.54)	73.20 (9.38)
	Male (%)	60.82	77.27
	Female (%)	39.18	22.73
	Rankin	1.09 (0.99)	1.59 (1.47)
	Diabetes (%)	81.44	86.36
	Non-Smoker (%)	47.42	38.1
	Ex-Smoker (%)	34.02	42.85
	Current Smoker (%)	18.56	19.05
	Treated Hypertension (%)	92.78	90.91
	Treated Hypercholesterolaemia (%)	85.57	86.36
	Average time to dementia onset (years)	–	3.02 (1.49)
	Average age of dementia onset (years)	–	76.23

MRI Parameters	Mean grey matter (mm^3^)	681,213 (76616)	670,374 (68616)
	Mean white matter (mm^3^)	333,930 (56698)	320,761 (56010)
	Mean total cerebral volume (mm^3^)	1,052,347 (114243)	1,036,447 (109535)
	Mean lacunar volume (mm^3^)	468 (671)	646 (661)
	Mean white matter hyperintesities volume (mm^3^)	37,204 (36937)	45,312 (30469)
	SVDp: Percentage ratio of WMH to GM volumes (%)	3.48 (3.01)	4.34 (2.99)

Cognitive	MMSE (Median and range)⁎⁎⁎	29 (22–30)	26 (16–30)
	NART⁎	100.03 (15.35)	93 (14.96)
	Executive function⁎⁎	−0.78 (1.07)	−1.35 (1.02)
	Processing speed	−0.83 (0.85)	−1.21 (0.95)
	Global functioning⁎	−0.49 (0.83)	−0.90 (0.82)
	Working memory	−0.16 (0.94)	−0.30 (0.93)
	Long-term memory	0.01 (0.98)	−0.44 (0.91)

Bonferroni corrected P < 0.003.

⁎ P < 0.05.

⁎⁎ P < 0.01.

⁎⁎⁎ P < .001.

### 3.2 Lacunar analysis

**Fig. 1:**
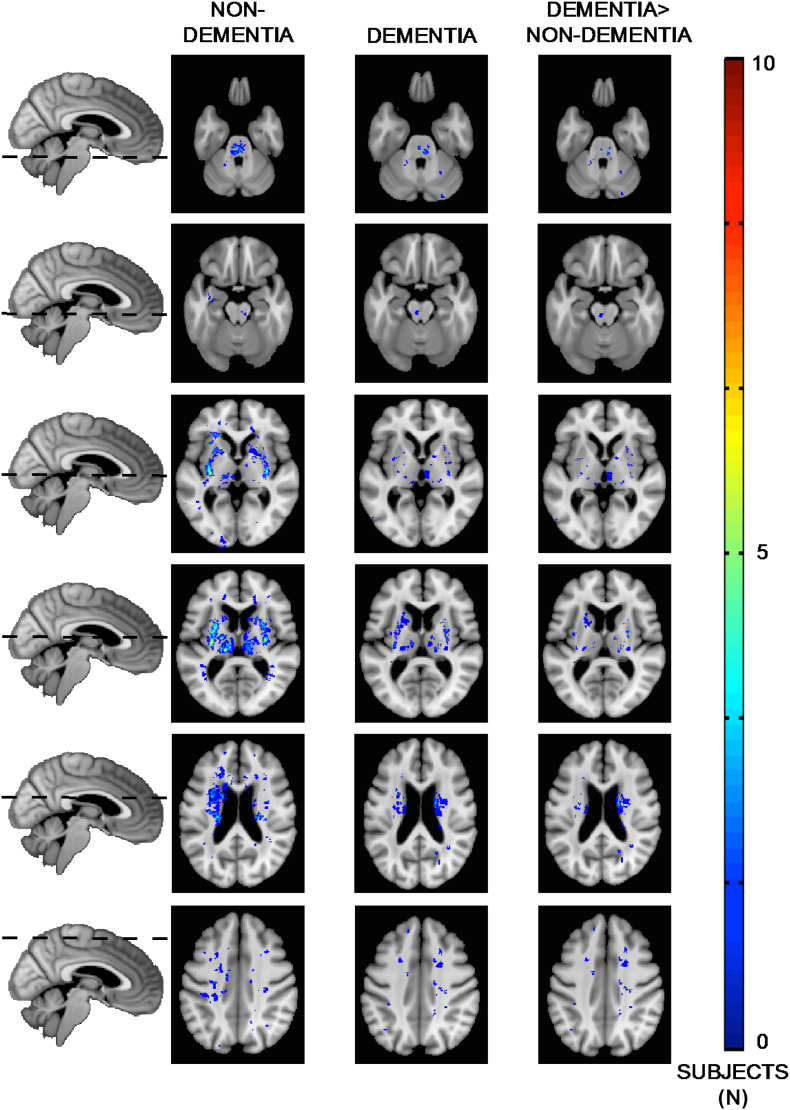
Distribution of lacunes between the preclinical dementia and non-dementing patient cohorts.

Differences in overlapping regions of lacune infarcts are shown in Fig. 1, and summarized in Supplementary Table 2. Whilst there were more lacunes present in the preVaD cohort (Table 1), this was not significant. When lacunes were present, they were more frequently located in the preVaD group (difference in the percentage of individuals with a lacune infarct greater or equal to 9%) particularly within in the striatum, corona radiata and internal capsule. Additionally, the preVaD cohort demonstrated greater asymmetry, with more lacunar infarcts located within the left-hemisphere. Whilst the number of thalamic lacunes were reasonably equal (32% preVaD, 30% normal), analysis of the sub-nuclei revealed more located (difference greater than or equal to 9%) within the ventro-anterior, mediodorsal, ventro-posterior and pulvinar in the preVaD group. Together, these findings indicate that a proportion of those with preclinical dementia already have strategic lacunes within structures associated with memory and language.

### 3.3 Voxel based morphometry

**Fig. 2:**
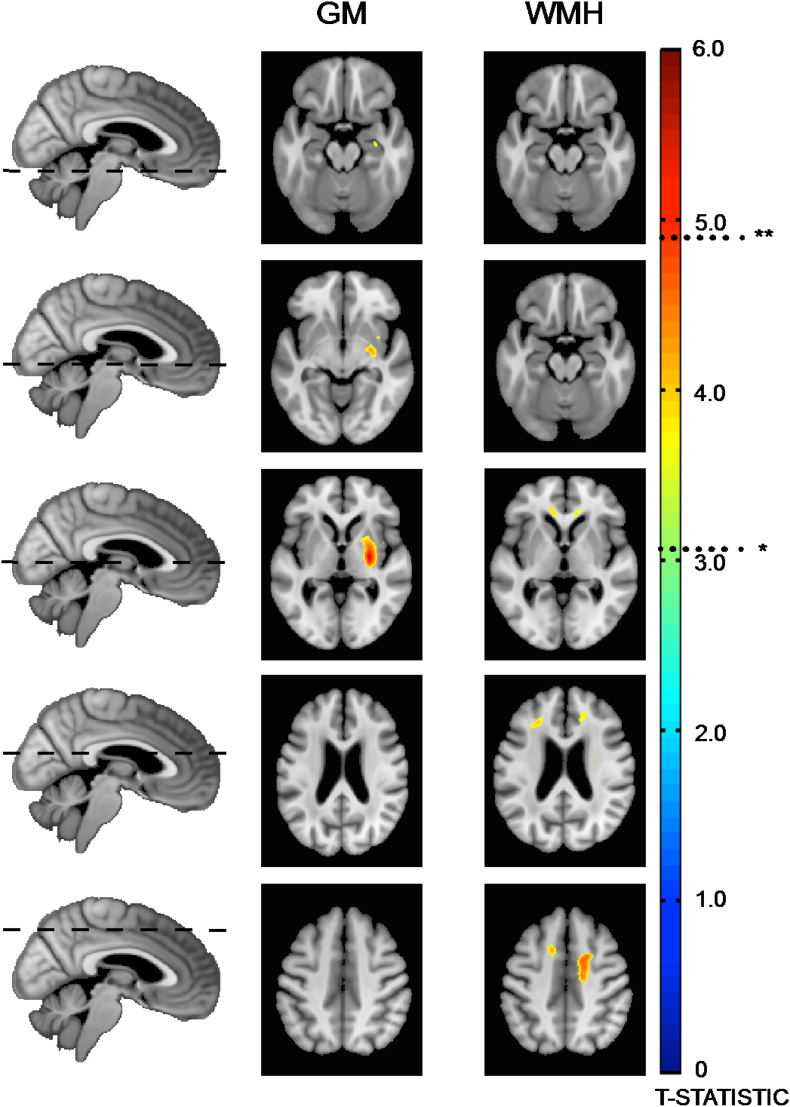
Voxel-based morphometry regions of decrease GM density and increased white-matter hyperintensities between the preclinical dementia and normal patient cohorts. *P < 0.001 uncorrected, **P < 0.05 FWE corrected.

Regions of significantly reduced grey matter density in the preVaD group are shown in Fig. 2. It demonstrates maked reduction in grey matter density within the left striatum, which is likely due to the distribution of lacunes. To a much lesser extent, reduced grey matter density is observed in the left anterior hippocampus and posterior portion of the right putamen.

Regions of significantly increased WMH were also found in the preVaD group, as shown in Fig. 2. It demonstrates more WMH within the frontal radiations. No significant WM changes were found (P < 0.05).

### 3.4 Cortical thickness

No significant regions were found (FWE P < 0.05).

### 3.5 Longitudinal rate analysis

No significant regions were found in the voxel wise analysis of the GM, WM or WMH rate maps (FWE P < 0.05).

### 3.6 Preclinical vascular dementia - SVM prediction

To better characterize the VBM analysis, we set out to test if future dementia could be predicted from voxel-wise, baseline MRI parameters using support vector machines. These results are summarized in Table 2. Overall, the most accurate predictions (judged by the balanced accuracy) were achieved using grey matter volumetric data. These appear to be particularly driven by the subcortical structures, with the striatum, hippocampus and to a lesser extent the thalamus, each able to independently predict future dementia (P < 0.01). The best predictions were achieved using combined striatal-hippocampal masks, providing a total prediction accuracy of 84.87%, balanced prediction accuracy of 73.17% (P < 0.01), class sensitivity 55% (P < 0.01), specificity 92% (P < 0.05) and an area under the receiver operator curve (c statistic) of 0.84.

**Table 2:** SVM predictions using baseline MRI data.

	Total accuracy %	Balanced accuracy %	Balanced accuracy *P* value	Class accuracy %		Class accuracy *P* value		Class predictive value %		ROC
				**Sensitivity**	**Specificity**	**Sensitivity**	**Specificity**	**Positive predictive value**	**Negative predictive value**	
Jacobian	83.19	63.33	0.01	31.82	94.85	0.01	0.82	58.33	85.98	0.79
GM	84.87	74.36	0.01	31.82	96.91	0.01	0.07	70	86.24	0.79
WM	79.83	52.48	0.1	9.09	95.88	0.24	0.12	33.33	82.3	0.62
WMH	79.83	54.24	0.01	13.64	94.85	0.01	0.95	37.5	82.88	0.65
Lacunar	84.03	56.82	0.01	13.64	100	0.02	0.06	100	83.62	0.64
Cortical Thickness	79.83	57.76	0.01	22.73	92.78	0.02	0.52	41.67	84.11	0.71
Putamen	82.35	68.09	0.01	45.45	90.72	0.01	0.04	52.63	88	0.78
Caudate	78.99	60.75	0.01	31.82	89.69	0.04	0.05	41.18	85.29	0.73
Striatum (Putamen + Caudate)	80.67	65.3	0.01	40.91	89.69	0.01	0.15	47.37	87	0.77
Hippocampus	83.19	65.09	0.01	36.36	93.81	0.02	0.01	57.14	86.87	0.8
Thalamus	80.67	61.79	0.01	31.82	91.75	0.01	0.01	46.67	85.58	0.72
Putamen + Hippocampus	84.87	69.93	0.01	45.45	93.81	0.01	0.01	62.5	88.35	0.84
Striatum + Hippocampus	84.87	73.17	0.01	54.55	91.75	0.01	0.05	60	89.9	0.84
Putamen + Hippocampus + Thalamus	83.19	70.36	0.01	50	90.72	0.01	0.15	55	88.89	0.81
Striatum + Hippocampus + Thalamus	82.35	66.33	0.01	40.91	91.75	0.01	0.15	52.94	87.25	0.81

The masked analysis used subcortical ROIs and the Jacobian determinant data. The best prediction (judged by balanced accuracy) was achieved using the combined striatum-hippocampus mask, which was significant at P < 0.01 using permutation testing (10,000 permutations)

### 3.7 Preclinical dementia – anatomical subgroups

**Fig. 3:**
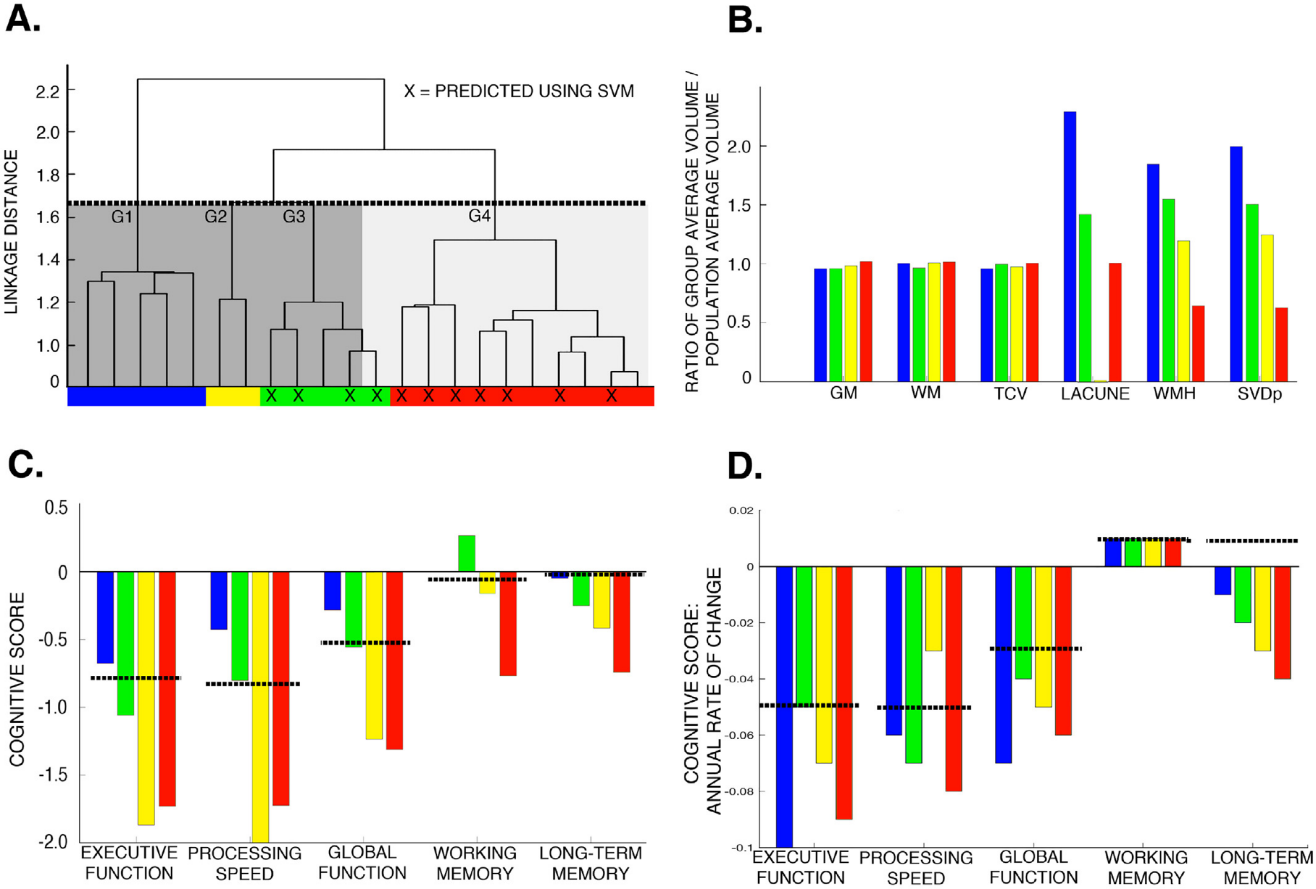
Anatomical subgroups in the preclinical dementia cohort. A) Dendrogram generated using ward-linkage on the unrepaired Jacobian determinant images. Dashed line shows the cut-off used to identify the four subgroups. The successful support vector machine predictions are shown (X), and the level of vascular damage indicated (dark grey - SVDp >4%; light grey – SVDp <4%). B) Differences in the MRI parameters between the identified sub-groups. These have been normalised by the population average to allow all parameters to be shown on a single plot. C) Differences in the cognitive parameters between the identified sub-groups, dashed line indicates population average. D) Differences in the rate of change in the cognitive profile, dashed line indicates population average.

Four subgroups were identified (Fig. 3, Table 3) on the basis of the dendrogram. Subgroups differed in the extent to which the SVM was able to correctly predict dementia. Examination of the negative prediction group revealed that the missed preclinical dementias all had significant vascular damage. By plotting the sensitivity and specificity curves over a range of SVDp, the optimal threshold for discriminating the negative prediction preclinical dementia group was SVDp = 4% (sensitivity = 64%, specificity = 68%), and therefore we used that as the definition for “high” and “low” vascular burden (also shown on Fig. 3).

**Table 3:** Baseline demographic, MRI and cognitive parameters for the dementia subgroups defined using cluster ward-linkage.

		GROUP 1	GROUP 2	GROUP 3	GROUP 4
Demographics	N	5	2	5	10
	Correctly predicted (%)	0	0	80	70
	Average age (years)	65.02 (10.67)	78.33 (9.90)	75.93 (7.97)	74.91 (7.23)
	Male (%)	60	50	60	70
	Female (%)	40	50	40	30
	Rankin	1.20 (1.30)	0.5 (0.71)	2.4 (1.82)	1.6 (1.43)
	MMSE	27.40 (2.97)	24.00 (4.24)	22.80 (4.82)	25.00 (3.13)
	NART	100.00 (14.93)	102.50 (33.23)	92.20 (12.36)	88.00 (12.53)
	Time to dementia onset (years)	3.87 (1.43)	2.82 (0.45)	2.66 (0.77)	2.83 (1.85)

MRI Parameters	Mean grey matter (mm^3^)	649,603 (84615)	667,153 (18905)	651,225 (73883)	690,978 (84449)
	Mean white matter (mm^3^)	281,955 (79245)	308,675 (34558)	331,980 (37052)	336,972 (52306)
	Mean total cerebral volume (mm^3^)	1,002,964 (132771)	1,022,045 (53432)	1,043,029 (106885)	1,052,777 (127415)
	Mean lacunar volume (mm^3^)	1137.60 (948.89)	5.50 (7.78)	706.20 (707.45)	499.00 (381.69)
	Mean white matter hyperintesities volume (mm^3^)	71,406 (26707)	46,218 (31.82)	59,824 (38959)	24,827 (14590)
	SVDp: Percentage ratio of WMH to GM volumes (%)	7.27 (3.04)	4.53 (0.24)	5.27 (3.40)	2.28 (1.16)

Cognitive	Executive Function	−0.68 (1.04)	−1.87 (0.15)	−1.06 (1.30)	−1.73 (0.53)
	Processing Speed	−0.43 (0.67)	−2.02^a^	−0.80 (1.24)	−1.72 (0.58)
	Global Functioning	−0.28 (0.69)	−1.24 (0.24)	−0.56 (1.14)	−1.31 (0.33)
	Working Memory	0.00 (0.78)	−0.17 (1.18)	0.27 (1.09)	−0.77 (0.75)
	Long-term Memory	−0.05 (1.10)	−0.42 (0.12)	−0.25 (1.26)	−0.74 (0.69)

a Only one data point available, other subject unable to complete task due to unrelated disability.

**Fig. 4:**
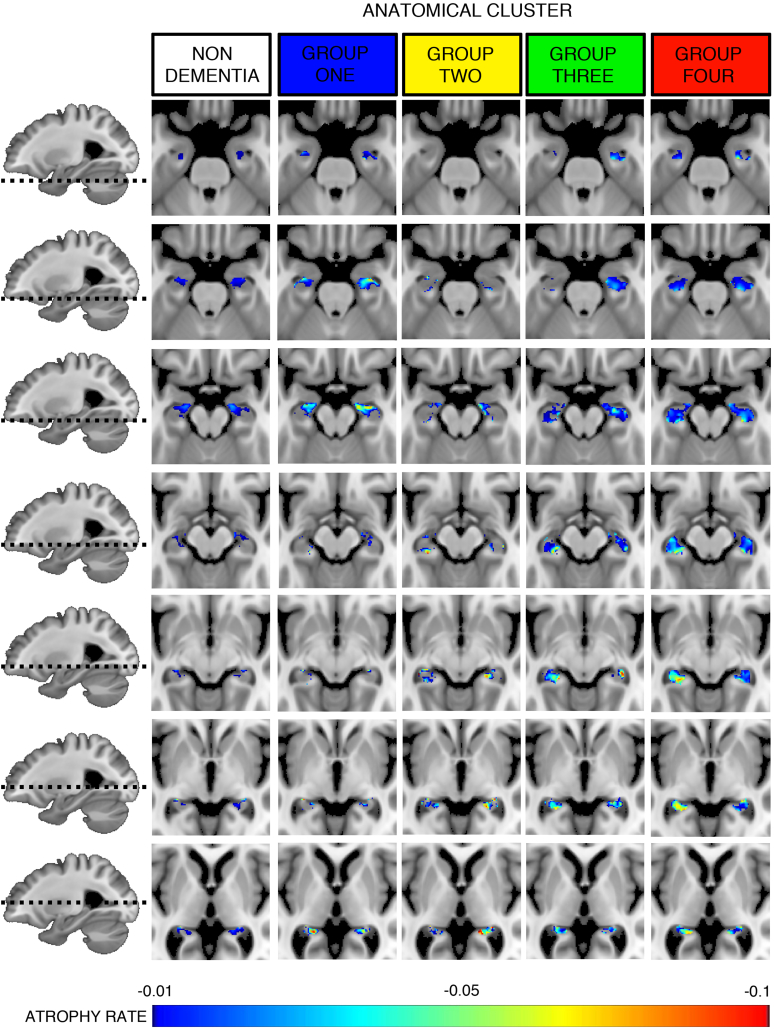
Longitudinal rate of hippocampal atrophy in anatomical subgroups.

In terms of the identified anatomical groups: Group 1 (n = 5) was associated with the highest burden of vascular disease, more cortical atrophy, a relatively normal baseline cognitive profile compared to the population average but more rapid deterioration in EF and PS, faster increases in SVDp and new lacunes, and a younger age of dementia onset. This pattern is consistent with primary vascular dementia (Kim et al., 2014), and could not be predicted through the best performing SVM analysis. Group 4 (n = 10) had the lowest level of vascular damage and whole brain atrophy, but poor baseline cognitive profile in all domains, notably working and long-term memory, and high levels of annual deterioration in the cognitive parameters but the lowest in the MRI SVDp parameters. This pattern is more Alzheimer's like, and 70% of cases could be predicted at baseline. Group 2 (n = 2) and group 3 (n = 5), which were more similar on the dendrogram, fell between these two extremes. Despite small numbers in each group, we aimed to further validate this observation by calculating the voxel-wise atrophy for the hippocampi (Fig. 4). This demonstrates that group 1 has a similar pattern of anterior hippocampal atrophy as the non-dementing cohort, where as group 4 is characterized by a much more global pattern. These observations are in keeping with previously described patterns of hippocampal atrophy in ageing and Alzheimer's (Frisoni et al., 2008).

### 3.8 Preclinical dementia – anatomical subgroups

**Fig. 5:**
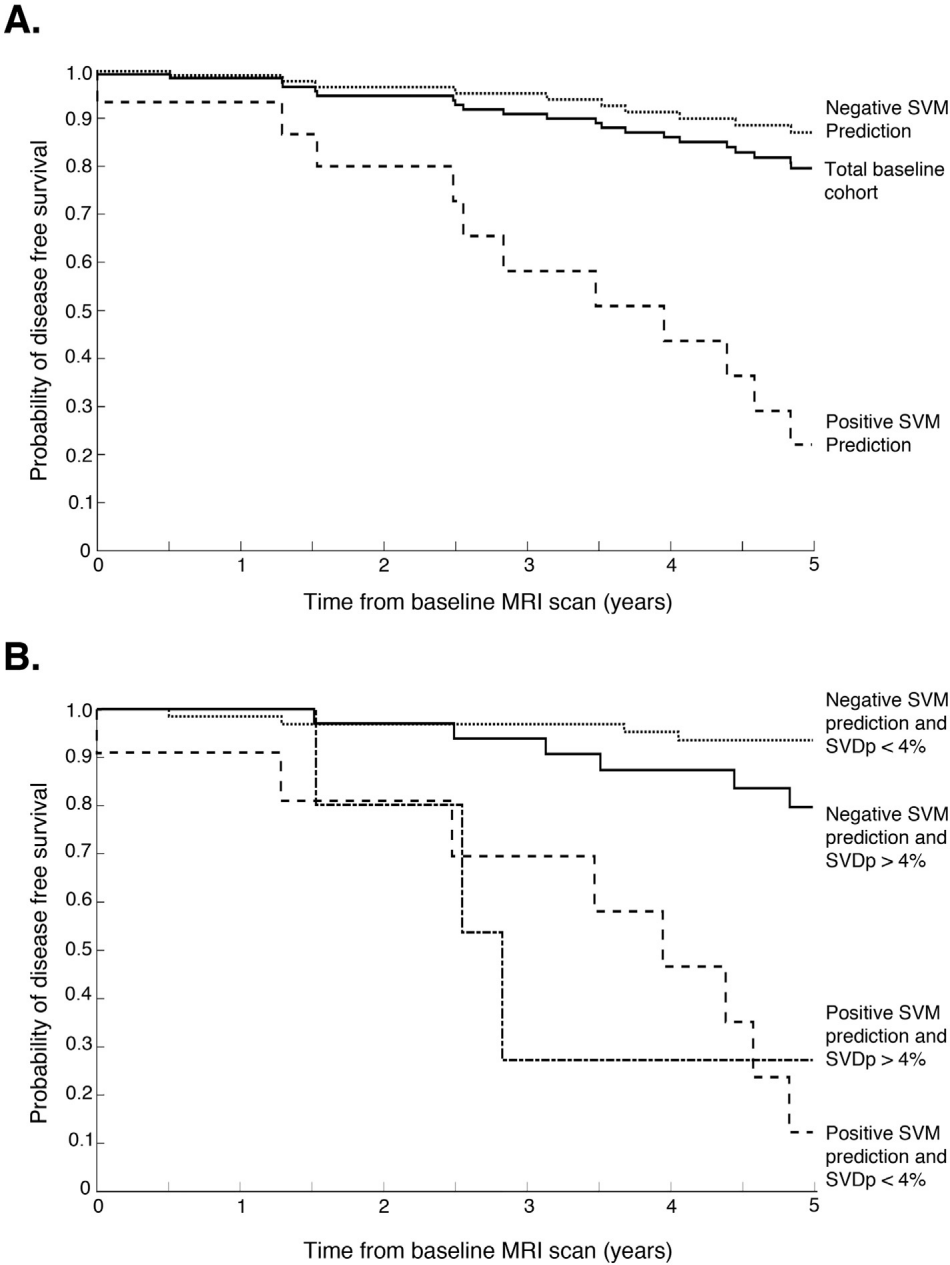
Kaplan-Meier survival plots for dementia onset. A. Total group risk plotted with positive and negative SVM predictions (log-rank P = 0.002). B. SVM predictions stratified according to vascular burden (Low - SVDp <4%; High – SVDp >4%; log-rank P = .005 between SVM prediction groups but not significant within groups).

The Kaplan-Meier survival curves are shown in Fig. 5. There is a significant difference between those predicted at baseline to develop dementia using SVM versus those not. The median survival time for the positive predictions is 3.5y (P < 0.005), corresponding to a 93% probability of survival in the negative prediction group. Due to the observed sub-group effect associated with high levels of vascular disease causing false negatives, the groups were stratified by vascular burden. Taking this into account (Fig. 4B), in those with a positive SVM prediction at baseline 73% with a low vascular burden and 60% with a high vascular burden will convert to dementia within 5y, compared to the negative predictions where 6% of the low vascular burden and 19% high vascular burden will develop dementia over the same timeframe.

## 4 Discussion

This study has demonstrated that within a cohort of patients with symptomatic cerebral small vessel disease there are anatomical abnormalities present in MRI during the preclinical phase of vascular dementia that can be used to accurately predict future dementia. Furthermore, we have demonstrated the existence of distinct pathological subgroups, based on anatomical differences detected using baseline MRI appearances, within those individuals who developed dementia during the five-year follow-up. These subgroups are consistent with a spectrum from a primary vascular dementia, with an extremely high burden of vascular disease, more rapid deterioration in EF and PS, and a younger age of dementia onset, that could not be predicted through SVM analysis, through to an older group with low level of vascular damage and whole brain atrophy, poor baseline cognitive profile in all domains, notably working and long term memory, with high levels of annual deterioration, reflecting a more Alzheimer's like pattern that could be predicted in 70% of cases. Two groups fell between these extremes and appear to represent a spectrum of mixed vascular and Alzheimer's like pathology (Fig. 4; Supplementary Fig. 2). Finally, we have shown that our prediction of dementia from baseline MRI data can be improved by accounting for the overall level of vascular damage, here defined as a SVDp more or <4%, which serves as a simple surrogate marker to stratify the aforementioned groups (i.e. 19% of individuals with negative predictions but SVDp >4% will develop dementia within 5y compared to 6% with SVDp <4%).

### 4.1 Structural correlates of preclinical vascular dementia

In this work, we found marked volumetric reduction in the left striatum and to a lesser extent anterior hippocampus. Associated with this, there were increased white-matter hyperintensities, particularly within the frontal radiations and left centrum semiovale, which has been previously observed in histologically confirmed vascular dementia (De Reuck et al., 2016). We also found an asymmetric distribution of lacunar damage, with more focal lesions within these areas in those with preclinical dementia that may account for some of these observations. In particular, 25% of subjects had focal, overlapping damage within the left centrum semiovale, which contains the superior longitudinal fasciculus and arcuate fasciculus. Therefore lesions in this area are more likely to cause disruption to the dominant hemisphere language networks. In a sub-analysis of the group 1 cluster, who had a primarily vascular dementia, we found that four out of the five patients had lacunes in this particular region. Whilst the numbers are small and should be interpreted with caution, these results suggest that this area, which has also been highlighted by others (De Reuck et al., 2016; Nestor et al., 2017), may be a strategic brain region where focal damage lowers the threshold to manifest symptoms of dementia via disruption of connecting networks (Nestor et al., 2017). Similarly, lesions particularly within the dominant hemisphere striatum and thalamic nuclei (medio-dorsal, ventro-anterior and pulvinar nuclei) were also more frequently observed in pre-clinical dementia, which is in line with previous work examining the role of strategic lacunes in cognitive impairment (Benisty et al., 2009; Benjamin et al., 2014) and overt vascular dementia. Importantly, we found no differences in the voxel-wise rates of WMH expansion or cortical atrophy between the groups, indicating that the location of ischaemic lesions are more important than the rate of SVD progression in the development of future dementia. Whilst the asymmetry of the results may at first appear unexpected, it is well known that many commonly used cognitive tests, such as the MMSE, are biased towards dominant left hemisphere function (Nelson et al., 1986; Kupke et al., 1993). Dementia is a clinically defined syndrome and therefore these results may just reflect a diagnostic bias, in that dominant hemisphere damage is simply easier to detect.

### 4.2 Subtypes of vascular dementia

One strength of this work is the use of strict inclusion criteria to generate a more homogenous cohort of symptomatic small vessel disease, defined as evidence of a lacunar infarct as well as confluent leukoaraiosis, but without evidence of large cortical infarcts, or other cause of white matter disease, together with a prolonged period of longitudinal follow-up. Rather than selecting patients presenting with cognitive impairment, which might favour recruitment of those with a mixed dementia we recruited patients with lacunar stroke regardless of the presence or absence of any cognitive impairment. At recruitment few had symptomatic cognitive complaints. Any subsequent clinically diagnosed dementia that developed within this group would automatically fulfill the DSM-V criteria (Gold et al., 1997) for vascular dementia. However, it is well established that at post-mortem in elderly individuals the underlying pathology is either pure Alzheimer's or mixed Alzheimer's with cerebrovascular disease (Kalaria, 2016), and only 5–7% will have a pure vascular dementia phenotype (Jellinger and Attems, 2010; Thal et al., 2012; Kalaria, 2016). The advent of amyloid PET imaging using the 11C-PiB ligand has allowed this to be better characterized in vivo by identifying individuals fulfilling the diagnostic criteria for vascular dementia without any evidence of amyloid pathology (Lee et al., 2011; Kim et al., 2014). These studies have shown that the cohorts with pure vascular dementia tend to be younger, with a greater burden of vascular damage as indexed by the number of lacunes.

In this work, we have identified four anatomical subtypes within a group that, as a minimum, has co-existing cerebrovascular disease to varying degrees (Fig. 5). The first group corresponds well with the descriptions of PiB negative pure vascular dementia, with a younger age of onset, extensive vascular damage (both WMH and LI) and brain atrophy, but with a cognitive profile that matches the more classical descriptions for a subcortical vascular dementia. This group could not be predicted using our SVM analysis. One possible explanation is that, given the pre-existing extensive lacunar damage, this group is at high risk for future lacunar events that may or may not be within a strategic location, but are inherently unpredictable. Additionally, the imaging modality used for this work may not have been sufficiently sensitive to detect the more diffuse damage that is present even in normal appearing white matter (Maillard et al., 2011). It may be that techniques such as Diffusion Weighted Imaging (DWI) may prove better at predicting this vascular subgroup by providing more accurate measures of these white matter abnormalities (Zeestraten et al., 2016), and already DWI has been shown to predict cognitive impairment in this patient group (Lawrence et al., 2014; Ciulli et al., 2016). In contrast, group four had very low levels of vascular disease, but marked memory impairments on cognitive testing, which would be more in keeping with Alzheimer's-like pathology (i.e. mixed Alzheimer's with associated cerebrovascular disease). The relatively predictable anatomical-clinical progression of this pathology also renders it easier to detect during the preclinical phase (Csernansky et al., 2005; Tondelli et al., 2012), and our classification parameters are similar to comparable work in preclinical Alzheimer's (Plant et al., 2010; Trzepacz et al., 2014). Furthermore, the observation that subcortical morphometric properties of the hippocampus, striatum, and to a lesser extent thalamus, provide much more accurate predictions in this AD-like group, align well with regional cortico-subcortical network disruptions that have previously been reported in AD associated with SVD (Nestor et al., 2017). The remaining two groups lay between these two extremes, with progressively decreasing amounts of vascular disease and increasing memory impairments.

The ability to reliably separate the two pathological extremes has several direct applications. First, it provides a quick, cheap, simple technique to predict and remove the majority of individuals with incipient Alzheimer's like pathology and provide an enriched cohort with “pure” vascular pathology where 19% will develop dementia within five years. It is likely that different therapies for Alzheimer's pathology and pure vascular pathology will be required, and mixing of the two pathologies may account for the mixed results achieved through previous clinical trials in vascular dementia (Kavirajan and Schneider, 2007; Skoog et al., 2012; Baskys and Cheng, 2012). Providing a way to better phenotype the baseline cohort will allow appropriately targeted clinical therapies, for example acetylcholinesterase inhibitors in the Alzheimer's cohorts versus aggressive blood pressure management in the vascular groups. Furthermore due to the simple nature of this technique, requiring only T1 weighted and FLAIR MRI images, it could be applied to pre-existing clinical trial datasets to investigate sub-group effects.

### 4.3 Limitations

The small group size of the preclinical vascular dementia cohort is a fundamental limitation to further sub-group analysis. Furthermore, there is no gold-standard post-mortem diagnosis for any of the patients, nor is there direct evidence for amyloid pathology in this work. Future work correlating the MRI results with either histology or PET imaging would be required to verify these findings. Based on previous histological studies it is extremely likely that the underlying pathology observed in the work is Alzheimer's, as supported by the subgroup rates of hippocampal atrophy (Fig. 4). However, due to the use of inferred (i.e. psychometric profiles, MRI parameters, rate maps) rather than direct evidence led us to adopt the term “*Alzheimer's like*” throughout the paper. Due to the inclusion criteria used, even those with a more “*Alzheimer's like*” pattern would be classified as mixed pathology (i.e. mixed Alzheimer's with associated cerebrovascular disease). However, our results allow this heterogeneous group to be stratified according to the likely predominant pathology, consistent with the latest recommendations (Custodio et al., 2017).

Post-diagnosis MRI scans were unavailable for the majority of our cohort, and therefore it is not possible to accurately define whether strategic lacunar events contributed to the final diagnosis. While diagnostic labels from the participant's routine clinical care are unavailable, but these are unlikely to have helped validate the proposed models as the discrepancy between clinical diagnosis by experts and post mortem can be as high as 20% (Archer et al., 2017), and standard clinical care would be unlikely to perform annual imaging and psychometric assessments at the same frequency as used in this work.

Whilst we did achieve classification parameters that were comparable with work in Alzheimer's (Csernansky et al., 2005; Tondelli et al., 2012), it may be possible to further improve on this by using the anatomical subtypes to refine cohorts into more phenotypically homogenous cohorts. However to achieve this, greater numbers would be required in each group. It may be that using MRI modalities that are more sensitive to white matter damage, such as diffusion weighted imaging, combined with more advanced classification methods that can combine multimodal data-sources (such as infinite kernel learning (Gehler and Nowozin, 2008)) would improve the classification parameters still further. However, achieving this requires substantial methodological development and is currently the focus of ongoing work.

It is acknowledged that our SVM analysis resulted in the best performing model using anatomical regions that are known to be involved in Alzheimer's disease rather than pure subcortical vascular dementia. Despite this, the agreement between these SVM results and anatomical clustering analysis are noteworthy. Despite the two approaches being independent of one another, they converge on a common solution and result in clinically coherent phenotypes that also agree with the broader literature, supporting the interpretation and proposed subgroups. Finally, whilst the focus of this work was to develop a technique that required only a single time-point to enable patient stratification, it may be that addition of further time-points may further improve the pre-clinical diagnosis.

## 5 Conclusion

Overall this work demonstrates that at a voxel-wise level, the pattern of disease is more important than the rate of progression in determining whether patients with SVD progress to dementia. We demonstrate that within a relatively homogenous cohort of cerebral small vessel disease, separate anatomical endophenotypes can be identified that correlate with long term clinical outcome, and that preclinical dementia in the groups with more Alzheimer's like pathology can be reliably predicted over three years before onset.

The following are the supplementary data related to this article.

Supplementary figure 1 Study Population Flow Chart. Supplementary figure 1

Supplementary figure 2 Proposed anatomical subgroups. Supplementary figure 2

Supplementary Table 1 Cognitive index scores. Supplementary Table 1

Supplementary Table 2 Summary of location of lacunar infarcts. Supplementary Table 2

Supplementary Material: Segmentation Methodology Image 1
