# Supplementary material for: Identifying preclinical vascular dementia in symptomatic small vessel disease using MRI
Source: Neuroimage Clin. 2018 Jun 20;19:925–38. doi: 10.1016/j.nicl.2018.06.023 (PMC6039843; doi:10.1016/j.nicl.2018.06.023)
Supplement: Supplementary figure 1 — Study Population Flow Chart. [file mmc1.docx]

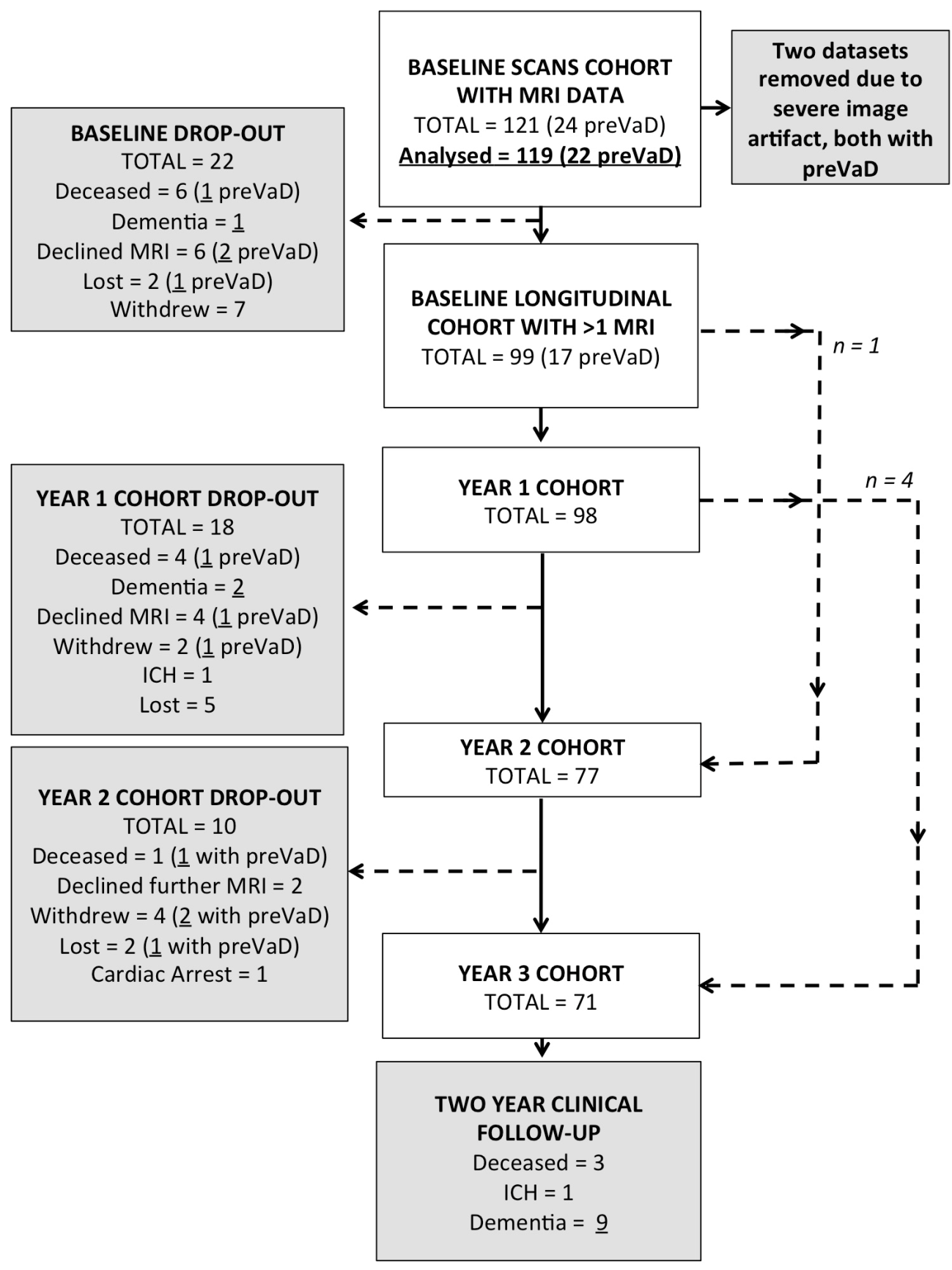


**Supplementary Figure 1:** Study Population Flow Chart. The preVaD group identified for this work are underlined for clarity. To note, the excluded baseline imaging datasets that were unsuitable for morphometric analysis were previously identified earlier work (Lambert et al 2015).
