# Supplementary material for: Identifying preclinical vascular dementia in symptomatic small vessel disease using MRI
Source: Neuroimage Clin. 2018 Jun 20;19:925–38. doi: 10.1016/j.nicl.2018.06.023 (PMC6039843; doi:10.1016/j.nicl.2018.06.023)
Supplement: Supplementary figure 2 — Proposed anatomical subgroups. [file mmc2.docx]

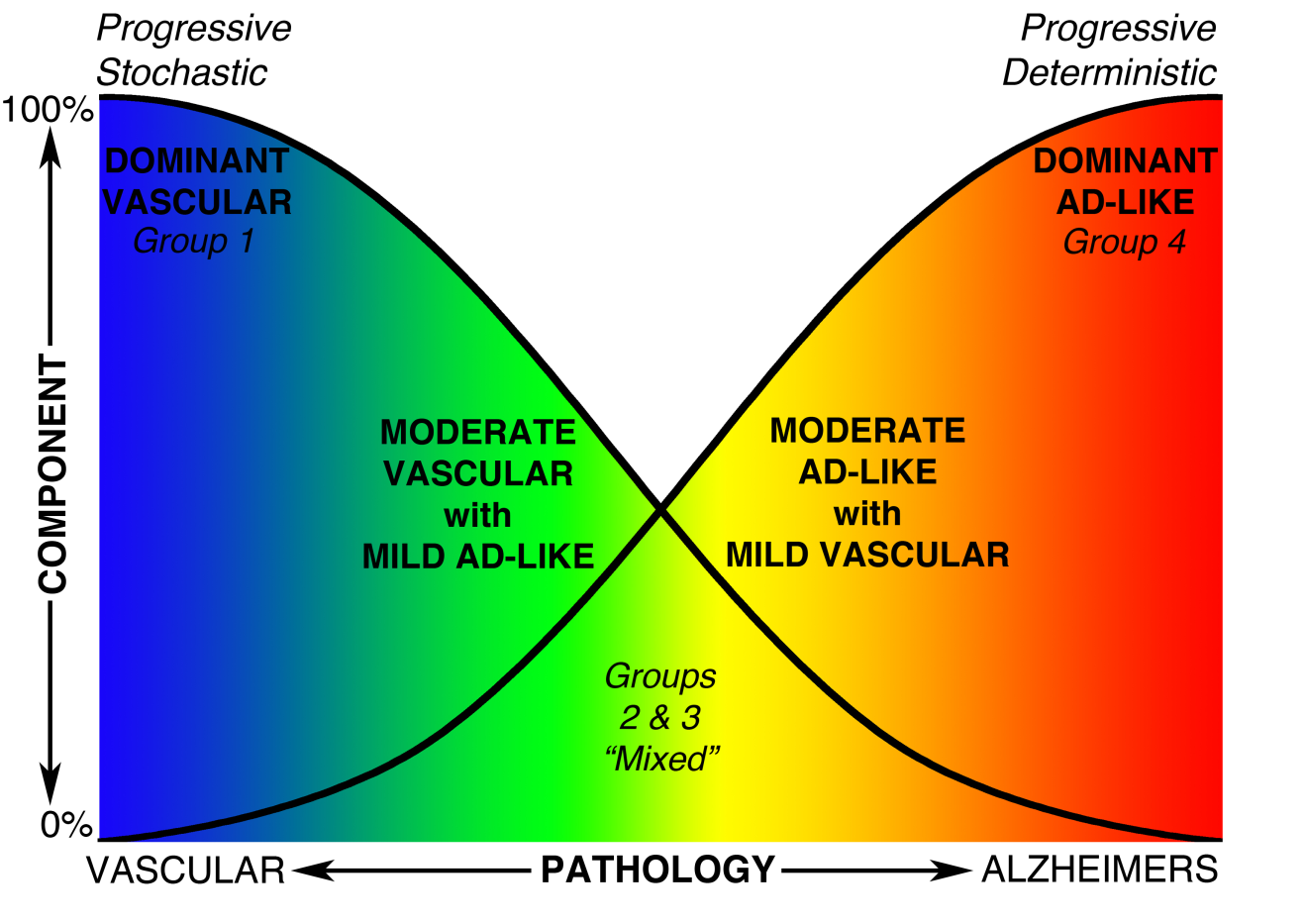


**Supplementary Figure 2:** Proposed anatomical subgroups. Group 1 was associated with an extremely high burden of vascular disease, more cortical atrophy, a relatively normal baseline cognitive profile compared to the population average but more rapid deterioration in executive function and processing speed, and a younger age of dementia onset. This pattern represents a primary vascular dementia, and could not be predicted through support vector machine analysis. This may reflect the stochastic nature of vascular damage. Group 4 had the lowest level of vascular damage and whole brain atrophy, poor baseline cognitive profile in all domains, notably working and long term memory, with high levels of annual deterioration. This pattern is more Alzheimer’s like, and 70% of cases could be predicted at baseline. This may reflect the gradually progressive nature of the disease. The remaining two groups fell between these two extremes and represent mixed pathology.
