# Supplementary material for: Identifying preclinical vascular dementia in symptomatic small vessel disease using MRI
Source: Neuroimage Clin. 2018 Jun 20;19:925–38. doi: 10.1016/j.nicl.2018.06.023 (PMC6039843; doi:10.1016/j.nicl.2018.06.023)
Supplement: Supplementary Table 1 — Cognitive index scores. [file mmc3.docx]

| Cognitive Index | Task & Normative Data reference | Task Measure(s) Used & Additional Details |
| --- | --- | --- |
| Executive Function (EF) | | |
|  |  | Time to complete Part B (number-letter switching) |
|  | Verbal Fluency | Total number of Correct Words generated |
|  | Modified Wisconsin Card Sort Test | Categories Achieved & Perseverative Errors* |
| Processing Speed (PS) | | |
|  | BMIPB Speed of Information Processing | Total correct, adjusted for motor score & errors (%)* |
|  | Digit Symbol Substitution | Total Correct |
|  | Grooved Pegboard Task | Time to complete (average of 2 hands) |
| Working Memory (WM) | | |
|  | Digit Span Task | Total Score |
| Long Term (Episodic) Memory (LTM) | | |
|  | WMS-III Logical Memory | Total Score: Immediate Recall & Delayed Recall* |
|  | WMS-III Visual Reproduction | Total Score: Immediate Recall & Delayed Recall* |

**Supplementary Table 1:** Cognitive index scores. BMIPB - Birt Memory & Information Processing Battery; WMS-III - Wechsler Memory Scale - Third Edition (UK). *Composite score used for multiple task measures. In addition a Global Cognition index was produced which summarised performance on all the tasks. Premorbid IQ was estimated using the re-standardised National Adult Reading Test (NART; 2^nd^ Edition).
