# Supplementary material for: Identifying preclinical vascular dementia in symptomatic small vessel disease using MRI
Source: Neuroimage Clin. 2018 Jun 20;19:925–38. doi: 10.1016/j.nicl.2018.06.023 (PMC6039843; doi:10.1016/j.nicl.2018.06.023)
Supplement: Supplementary Table 2 — Summary of location of lacunar infarcts. [file mmc4.docx]

**Supplementary Table 2:** Summary of location of lacunar infarcts (percentage of individuals within each group)
