# Supplementary material for: Identifying preclinical vascular dementia in symptomatic small vessel disease using MRI
Source: Neuroimage Clin. 2018 Jun 20;19:925–38. doi: 10.1016/j.nicl.2018.06.023 (PMC6039843; doi:10.1016/j.nicl.2018.06.023)
Supplement: Supplementary file 5 — Supplementary Material: Segmentation Methodology [file mmc5.docx]

**Supplementary Material: Segmentation Methodology**

Further details into the segmentation methodology are provided here. A detailed explanation may also be found in Lambert *et al.,* 2016 with technical details for generation of population specific TPMs in Lambert *et al.,* 2013. For clarity, SPM8 New Segment was used to develop and apply the segmentation pipeline (as this version was available during initial methodological development) but with customized TPMs generated from our data rather than default SPM TPMs. SPM12 was available for our later analysis and was used for all subsequent longitudinal warping (via the new “*Shoot*” toolbox) and statistical analysis.

**Segmentation Step 1 – Create Initial Group Average Space**

Initially the native space T1-weighted images were segmented into GM, WM and CSF tissue classes using the unified segmentation (“*New Segment*”) within SPM8 (Ashburner and Friston, 2005). These segmentations were registered to a common group-average 1mm isotropic voxel template image using a diffeomorphic registration algorithm (Ashburner and Friston, 2011), and the T1-weighted and FLAIR images warped to this space. These images were skull-stripped using the warped segmentations at a tissue probability threshold of 0.1, and manually checked and corrected where necessary.

**Segmentation Step 2 – Create Population Specific Tissue Probability Maps**

Population specific tissue probability maps (TPMs) were created for three tissue classes (GM, WM and CSF) using the warped, skull-stripped T1-weighted images and a Modified Mixture of Gaussians algorithm to account for anatomical features specific to the study cohort, such as larger ventricles. Using the warped skull-stripped FLAIR and T1-weighted images, TPMs were generated for the white matter hyperintensities (WMH) using a Modified Multivariate Mixture of Gaussians (Lambert *et al.,* 2013). These TPMs then replaced the default maps supplied by SPM in the *New Segment* pipeline.

**Segmentation Step 3 – Re-segmenting the Native Space Images**

The new TPMs were used to re-segment the native space structural images into grey matter, white matter, CSF and WMH. The central corpus callosum and fornix were excluded from the WMH segmentation by masking with a group average mask. This was performed to reduce the regional variation of voxel intensities in these structures that cause frequent tissue misclassification. The WMH segmentations were binarised at a manually determined threshold for each individual. This threshold was determined by overlaying the segmentations onto the corresponding FLAIR images using ITK-SNAP version 2.2.0 (www.itksnap.org) (Yushkevich *et al.,* 2006), to ensure each individual WMH map accurately defined the spatial extent of the lesions. This step was performed independently by two raters (CL and EZ), and inter- and intra-rater reliability metrics checked using 10 randomly selected scans across all time points, duplicated to provide a total set of 20 scans per rater, each set randomly re-ordered for each rater to avoid any sequence bias. Both raters were blinded with respect to subject and time-point of each scan. Each binary map was further checked and corrected where necessary using ITK-SNAP. The WMH manual refinement removed areas of mis-segmentation that were apparent in some areas with similar voxel intensity profiles to WMHs, and was most often required in the hippocampus and posterior insula. This step did not alter the contours or extent of the automatically delineated WMH lesions. Areas of lacunar infarction were manually identified on native space T1-weighted images using published criteria (Benjamin *et al.,* 2014). This was aided by overlaying CSF segmentations on corresponding FLAIR images to identify regions of misclassification. Regions were segmented using a space-filling algorithm in ITK-SNAP, using voxel intensity thresholds (set to between 350 and 500) with 500 iterations. These results were visually checked and manually refined where necessary. This step was performed independently by two raters (CL and PB), and inter- and intra-rater reliability checked using 20 randomly selected scans across all time points as detailed above. Again the raters were blinded to the subject and time-point of each scan.

**Segmentation Step 4 – Tissue Repair**

One of the problems associated with segmenting brains with WMH and LI is that regions of damage can be misclassified into erroneous tissue types due to changing tissue intensity profiles as a result of pathology. Regions of WMH are often classed as grey matter, and gliosis within LI as CSF, in a standard segmentation result from SPM8. This misclassification will cause inaccuracies when warping the segmentations together to a group average template, and impair the ability to accurately map areas of damage to a common space. For example, a region of gliosis caused by a lacune in the head of the caudate would be warped with the CSF tissue class into the adjacent lateral ventricle. To overcome this limitation a segmentation repair step was undertaken. The WMH binary maps were used to identify brain regions that should be classed as WM. These regions were subsequently corrected in the corresponding WM and GM segmentations, by zeroing the WMH regions in GM, and adding them to WM. As LI may cross tissue boundaries they were mapped to the initial group average space and the TPMs were used to define the most likely tissue class in the LI regions over all time-points. This was then used to reclassify the native GM, WM and CSF maps.

To further improve the segmentations, each baseline T1-weighted image was processed using the recon-all pipeline in Freesurfer (version 5.0, (Fischl *et al.,* 2002)). The resultant parcellation image was binarised and combined with the repaired segmentations to create an individual brain mask. Each mask was then manually checked in ITK-SNAP to remove any erroneously classified brain voxels around the margins of the skull and dura. Copies of individual baseline brain masks were then co-registered using SPM12 to all future time-points using the corresponding T1-weighted images. The final brain mask for each time-point was then used to remove any erroneously classified grey and white matter voxels in the segmentations and these were included in the CSF images. At each time-point, the final result was visualised and, if necessary, the masks were further refined manually.

For each scan, the unrepaired segmentations were used to calculate tissue volumes. The WMH, LI, total cerebral volume (TCV, defined as the sum of grey matter, white matter and WMH at a tissue probability threshold ≥ 0.2) and total intracranial volume (TIV, defined as TCV plus the CSF volume at a set tissue probability threshold ≥ 0.2, constrained by the final brain inclusion mask) were calculated in mm^3^.

Reliability metrics were calculated as per Dunn *et al.,* 2014. The inter-rater reliability metrics were calculated using the mean values for each rater. Good measures of reliability were obtained for the MRI measures that needed manual refinement. For WMH, the inter-rater reliability metrics were: Standard error of mean = 247mm^3^, mean variability = 7.78% (standard deviation = 3.03%), Pearson’s intra-class correlation coefficient = 0.98. The intra-rater reliability metrics were: Standard error of mean = 187mm^3^, mean variability = 5.57% (standard deviation = 3.99%), Pearson’s intraclass correlation coefficient = 0.99. For LI, the inter-rater reliability metrics were: Standard error of mean = 3mm^3^, mean variability = 7.93% (standard deviation = 4.89%), Pearson’s intra-class correlation coefficient = 0.99. The corresponding intra-rater reliability metrics were: Standard error of mean = 2mm^3^, mean variability = 4.32% (standard deviation = 4.19%), Pearson’s intraclass correlation coefficient = 0.99. There were no significant differences between the volumes measured by the two raters.

**Longitudinal Warping Step 1 – Individual average space**

Initially for each individual, the five tissue type maps (grey matter, white matter, CSF, WMH and LI), and all available time-points (between two and four per subject) were warped to an individual average template using the Shoot toolbox in SPM12. This produced Jacobian determinant, warp and velocity fields for each time-point in addition to the individual average tissue template images. The velocity fields were used to calculate divergence maps using the in-built SPM12 function *spm_shoot_divergence*. For each voxel, the divergence maps quantify the diffeomorphic distance between the subject time-point image from the average image, and represent the amount of expansion or contraction required to warp it to the average image. For each voxel, across all divergence maps for an individual, a linear fit was calculated with respect to the time between scans to generate a single “*rate map*” for each subject. These steps were then repeated using the three repaired tissue classes to produce a repaired individual average image and a rate map for each corrected tissue type.

**Longitudinal Warping Step 2 – Group average space**

Individual repaired average tissue class templates were generated by the longitudinal warping in step 1. In step 2, the three average tissue classes for each individual were used in the Shoot pipeline to provide deformations to a group average space.

**REFERENCES:**

Ashburner J, Friston KJ. Unified segmentation. NeuroImage 2005; 26(3): 839-51.

Ashburner J, Friston KJ. Diffeomorphic registration using geodesic shooting and Gauss-Newton optimization. NeuroImage 2011; 55(3): 954-67.

Ashburner J, Ridgway GR. Symmetric diffeomorphic modeling of longitudinal structural MRI. Frontiers in neuroscience 2012; 6.

Dunn JT, Clark-Papasavas C, Marsden P, Baker S, Cleij M, Kapur S, et al. Establishing test-retest reliability of an adapted [^18^F]fallypride imaging protocol in older people. Journal of Cerebral Blood Flow & Metabolism 2013; 33(7): 1098-1103

Lambert C, Lutti A, Helms G, Frackowiak R, Ashburner J. Multiparametric brainstem segmentation using a modified multivariate mixture of Gaussians. NeuroImage: Clinical 2013; 2: 684-94.

Lambert C, Janakan SN, Barrick TR, Markus HS. Characterising the Grey Matter Correlates of Leukoaraiosis in Cerebral Small Vessel Disease. NeuroImage: Clinical 2015: 9: 194-205*.*

Lambert, C., Benjamin, P., Zeestraten, E., Lawrence, A.J., Barrick, T.R. and Markus, H.S., 2016. Longitudinal patterns of leukoaraiosis and brain atrophy in symptomatic small vessel disease. *Brain*, p.aww009.

Yushkevich PA, Piven J, Hazlett HC, Smith RG, Ho S., Gee JC, et al. User-guided 3D active contour segmentation of anatomical structures: significantly improved efficiency and reliability. NeuroImage 2006; 31(3): 1116-28.
